# Observational study on implications of the COVID-19-pandemic for cardiopulmonary resuscitation in out-of-hospital cardiac arrest: qualitative and quantitative insights from a model region in Germany

**DOI:** 10.1186/s12873-022-00628-2

**Published:** 2022-05-18

**Authors:** Domagoj Damjanovic, Jan-Steffen Pooth, Rebecca Steger, Martin Boeker, Michael Steger, Julian Ganter, Tobias Hack, Klemens Baldas, Paul Marc Biever, Daniel Schmitz, Hans-Jörg Busch, Michael Patrick Müller, Georg Trummer, Bonaventura Schmid

**Affiliations:** 1 Department of Cardiovascular Surgery, University Heart Center Freiburg, Faculty of Medicine, University of Freiburg, Hugstetter Str. 55, 79106 Freiburg, Germany; 2 Department of Emergency Medicine, Faculty of Medicine, University Hospital of Freiburg, University of Freiburg, Sir-Hans-A.-Krebs-Str, 79106 Freiburg, Germany; 3 Institute of Medical Biometry and Statistics, Medical Center - University of Freiburg, Faculty of Medicine, University of Freiburg, Stefan-Meier-Str. 26, 79104 Freiburg, Germany; 4 Department of Anaesthesiology, Intensive Care, and Emergency Medicine, St. Josef’s Hospital, Sautierstr. 1, 79104 Freiburg, Germany; 5 Department of Medicine III: Interdisciplinary Medical Intensive Care, Medical Center - University of Freiburg, Faculty of Medicine, University of Freiburg, Hugstetter Str. 55, 79106 Freiburg, Germany

**Keywords:** Cardiopulmonary resuscitation, Cardiac arrest, COVID-19, SARS-CoV-2, Chain of survival, Bystander CPR

## Abstract

**Background:**

The city of Freiburg has been among the most affected regions by the COVID-19 pandemic in Germany. In out of hospital cardiac arrest (OHCA) care, all parts of the rescue system were exposed to profound infrastructural changes. We aimed to provide a comprehensive overview of these changes in the resuscitation landscape in the Freiburg region.

**Methods:**

Utstein-style quantitative data on OHCA with CPR initiated, occurring in the first pandemic wave between February 27th, 2020 and April 30th, 2020 were compared to the same time periods between 2016 and 2019.

Additionally, qualitative changes in the entire rescue system were analyzed and described.

**Results:**

Incidence of OHCA with attempted CPR did not significantly increase during the pandemic period (11.1/100.000 inhabitants/63 days vs 10.4/100.000 inhabitants/63 days, *p* = 1.000). In witnessed cases, bystander-CPR decreased significantly from 57.7% (30/52) to 25% (4/16) (*p* = 0.043). A severe pre-existing condition (PEC) was documented more often, 66.7% (16/24) vs 38.2% (39/102) there were longer emergency medical services (EMS) response times, more resuscitation attempts terminated on scene, 62.5% (15/24) vs. 34.3% (35/102) and less patients transported to hospital (*p* = 0.019). Public basic life support courses, an app-based first-responder alarm system, Kids Save Lives activities and a prehospital extracorporeal CPR (eCPR) service were paused during the peak of the pandemic.

**Conclusion:**

In our region, bystander CPR in witnessed OHCA cases as well as the number of patients transported to hospital significantly decreased during the first pandemic wave. Several important parts of the resuscitation landscape were paused. The COVID-19 pandemic impedes OHCA care, which leads to additional casualties. Countermeasures should be taken.

**Supplementary Information:**

The online version contains supplementary material available at 10.1186/s12873-022-00628-2.

## Background

The city of Freiburg/Germany and its surrounding suburbs cover an area of 151.3 km^2^ with over 230.000 inhabitants [1]. Response to out of hospital cardiac arrest (OHCA) calls is based on a two-tiered rescue system with (A) paramedic-staffed ambulances, equipped according to European standard Mobile ICU, DIN EN 1789 (type C) to initiate Advanced Life Support; and (B) a physician staffed emergency response vehicle equipped according to German standard DIN 75079.

Recently, additional services have been implemented within this area in order to improve survival following out of hospital cardiac arrest (OHCA) considering this region as a model region of out-of-hospital cardiopulmonary resuscitation (CPR) care. These efforts include e.g. an smartphone-based alarm system (SAS) for trained rescuers (“Region der Lebensretter”), “KIDS SAVE LIVES” program *Löwen Retten Leben* [2], an out-of-hospital program for extracorporeal CPR (eCPR), certified Cardiac Arrest Center and implementation of an 24/7 cardiac arrest receiving team (CART) to improve postresuscitation care [3, 4].

The city of Freiburg has been among the most affected regions by the first COVID-19 pandemic wave in Germany [5], and is located in immediate vicinity to the most severely struck region Grand Est in France. Until April 30th, 2020 the local public health department registered 951 confirmed SARS-CoV-2 infections and 69 deaths in the city of Freiburg, resulting in a case-fatality-rate of 7.3%.

The federal state of Baden-Wuerttemberg announced its official “Corona regulation” on March 17th 2020. These regulations focus to minimize public life in general, closed all schools and multiple other places and called on everyone to reduce social contacts [6]. On March 19th 2020 the city of Freiburg intensified these regulations and announced a prohibition to access all public places for 2 weeks starting on March 21st, 2020. Therefore in OHCA care, all parts of the „chain of survival “were exposed to these infrastructural changes with an altered availability of resources and services, an increased need for staff protection and specific changes of medical management due to the ongoing pandemic. The consequences of such pandemic related changes of the health system have been described by Baldi et al. in the care of OHCA patients in Northern Italy [7, 8]. This group reported an 58% increase in OHCA cases compared to the same period in 2019. Unwitnessed arrests, arrests at home and arrests from medical causes increased in Northern Italy, while bystander-CPR rates dropped by over 15% [8]. Emergency medical services (EMS) arrived 3 min later, and the out-of-hospital death rate was almost 15% higher [7]. Similar outlines have been published for the Paris region [9]. The European Resuscitation Council (ERC) released COVID-19 Guidelines On April 24th,2020 [10]. Based on the recommendations of the International Liaison Committee on Resuscitation, the GRC also published a statement with updated recommendations for basic and advanced life support [11].

In the context of these profound changes due to the pandemic and given the complex nature of OHCA care with different involved institutions and influencing factors, we aimed to provide a comprehensive overview of changes to the resuscitation landscape in the Freiburg region.

## Methods

Quantitative data as well as qualitative information on pandemic-related changes OHCA care in our rescue system was collected and analyzed, to inform targeted interventions as well as measures of preparedness for future pandemic episodes.

### Setting

EMS region of Freiburg, Germany.

### Population

Patients with OHCA where CPR was initiated, as documented in prehospital EMS treatment protocols, were included in the quantitative analysis.

### Time frame

The first SARS-CoV-2 infection in the region of Freiburg was confirmed on February 27th, 2020. Therefore the corresponding time frame for this data work-up ranged from February 27th, 2020 until April 30th, 2020. The cases were compared to cases of OHCA in the same time periods between 2016 and 2019. To account for leap years, a time frame of 63 days in each year starting on February 27th was included.

### Data collection

Collection of quantitative data was performed in Utstein-style as internationally recommended and comprehensively described by Perkins et al. in 2015 [12]. It included information from the EMS dispatch center, prehospital emergency physician’s documentation and all patient receiving hospitals.

Incidence of OHCA was calculated while taking into account changes in population as reported by the local electoral office. It is presented in conjunction with infection numbers of SARS-CoV-2-infections in the region of Freiburg. Data regarding SARS-CoV-2 infections in the region of Freiburg were collected from the local public health department, which monitored and reported all infections with SARS-CoV-2. Regarding bystander CPR rates, a by-year analysis of the before mentioned timeframes was performed additionally. The data source for the mortality rate in the region are the local public health services. Although there is a close collaboration and a high common interest in data processing regarding impact of the pandemic, the mortality rate was not available for the exact time period used in this analysis.

Qualitative information was retrieved via personal correspondence with the heads or coordinators of the different parts of the rescue system, i.e.: the medical director of the EMS; the program coordinator of the federal state-wide kids save lives program; the program coordinator of the smartphone alerting system for trained rescuers; the program coordinators of the eCPR program. The most current regulatory statements regarding pandemic-related contact restrictions were retrieved from the webpage of the Federal State Government of Baden-Württemberg.

All ambulances in Freiburg use tablets for electronic documentation (MEONA Emergency Ambulance Software, MEONA GmbH, Freiburg, Germany) for all out-of-hospital cases. This documentation includes Utstein style elements as well as other medical information. The item “preexisting condition” allows for categorization of the patient’s status prior to the emergency, taking into account comorbidities and functional status with regard to independent living, as well as any progressive detioration, leaving room for an additional subjective evaluation by the emergency physician. Further items cover the response time (time from call to first vehicle on scene) and overall process times, details on patient management and progress during prehospital emergency care and transport.

The resulting reports are securely stored and made available as protocols to the treating hospitals via single-use patient unique identifiers. These datasets were searched for cases of OHCA in the Freiburg region since January 1st, 2016 with “CPR attempted” as the identifying data point, and the, Utstein elements from these datasets were entered into and managed using REDCap (Research Electronic Data Capture) tools hosted at the University Medical Center Freiburg. REDCap is a secure, web-based software platform designed to support data capture for research studies [13, 14].

### Statistical analysis

Continuous data are presented as mean with standard deviation or median with interquartile range, depending on distribution. Categorical data are presented as n in percent of total (%). Continuous variables were checked for normal distribution by Shapiro-Wilk test and afterwards compared with Student’s t test or Mann-Whitney U test as appropriate. Categorical variables were compared using Fisher’s exact test due to the sample size.

Statistical analysis was performed with RStudio software, version 1.2.1335. *p* values less than 0.05 were considered statistically significant.

### Ethical approval and trial registration

The retrieval and analysis of OHCA data has been approved by the ethics committee of the Albert-Ludwigs University Freiburg as part of an observational evaluation of the smartphone alerting system (SAS) for trained rescuers (Ethics committee approval number 482/18), as well as an observational evaluation of the out-of-hospital eCPR program (Ethics committee approval number 481/18). Therefore, no informed consent was required. Both studies are registered in the German registry for clinical studies (DRKS00016625 and DRKS00016627). All methods were carried out in accordance with relevant guidelines and regulations.

## Results

**Fig. 1:**
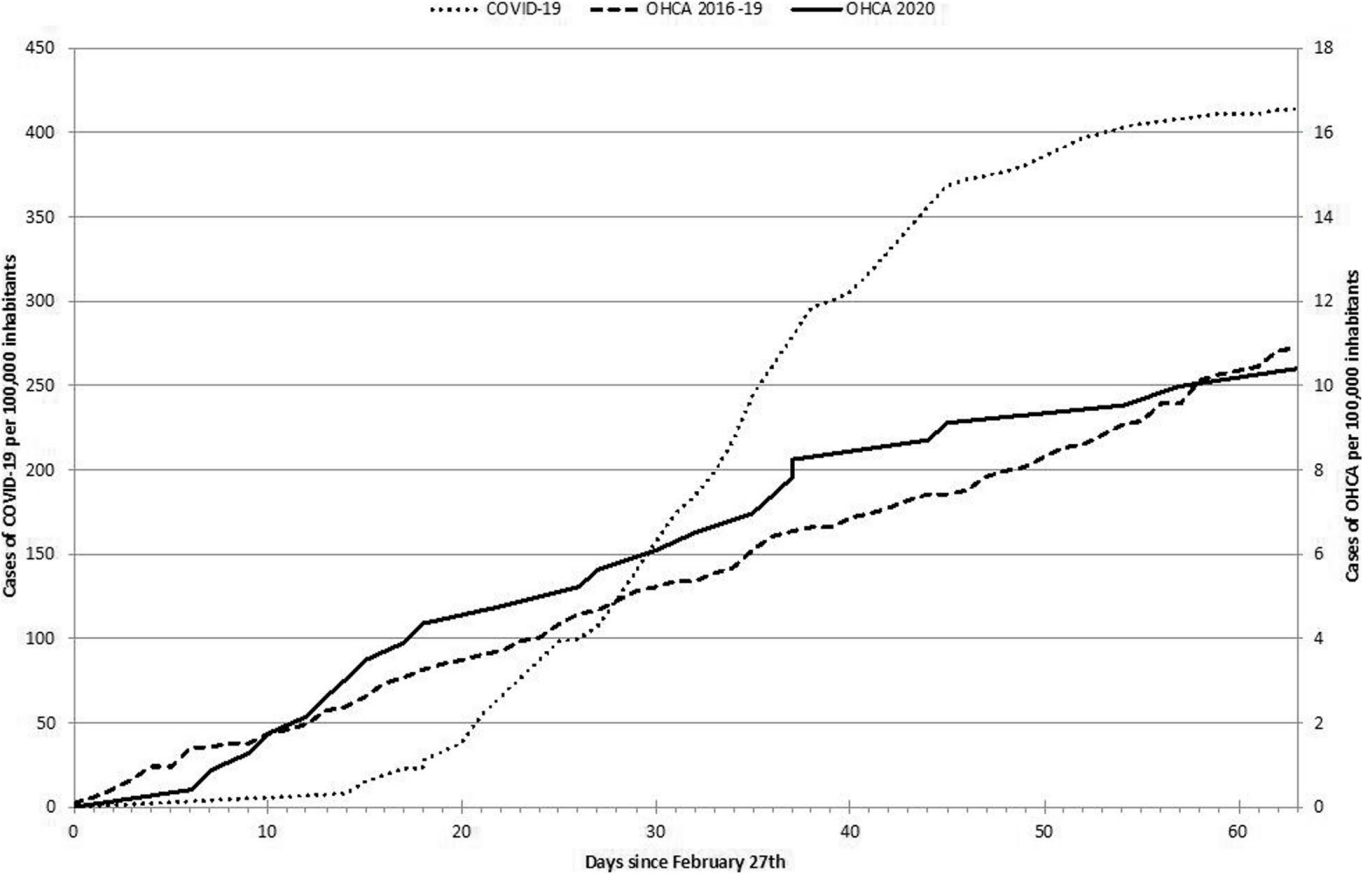
Incidence of COVID-19 (dotted line) vs incidence of OHCA in the same time frame of 2020 (solid line) compared to the years 2016–2019 (interrupted line). Incidence of OHCA did not significantly differ in 2020 compared to the previous years (*p* = 1)

Compared to the same periods in the non-pandemic years, incidence of OHCA with attempted CPR did not significantly increase during the defined pandemic period (11.1/100.000 inhabitants/63 days vs 10.4/100.000 inhabitants/63 days, *p* = 1.000, see Fig. 1 and Table 1). For the non-pandemic periods, there were *N* = 33 cases of OHCA in 2016, *N* = 20 in 2017, *N* = 20 in 2018 and *N* = 29 in 2019 registered in the database. Regarding the pandemic period of 2020 *N* = 24 cases of OHCA with attempted CPR were registered. Characteristics of OHCA cases are shown in Table 1.

**Table 1:** Characteristics of OHCA

	2016–2019	2020	*p*-value
System			
Cases of OHCA, Resuscitation attempted	102	24	n.a.
Incidence (cases of OHCA with CPR attempted/100.000 inhabitants/63 days)	11.1	10.4	1
Dispatch			
Dispatcher-identified CA*	42/69 (60.9%)	14/24 (58.3%)	1
Dispatcher CPR instructions*	7/69 (10.1%)	2/23 (8.7%)	1
Patient			
Age, years	68.9 (57.4; 79.7)	67.9 (58.7; 84.2)	0.7162
Sex			0.6396
Male	68/101 (67.3%)	15/24 (62.5%)	
Female	33/101 (32.7%)	9/24 (37.5%)	
Pre-existing conditions (PEC)			0.0214
No PEC / PEC without significant impact on daily life	51/102 (50.0%)	5/24 (20.8%)	0.0117
PEC with significant impact on daily life / normal daily life impossible	39/102 (38.2%)	16/24 (66.7%)	0.0208
PEC not documented	12/102 (11.8%)	3/24 (12.5%)	
witnessed CA	52/99 (52.5%)	16/24 (66.7%)	0.2564
CPR initiated by bystander	43/99 (43.4%)	8/24 (33.3%)	0.4894
CPR initiated by bystander when CA witnessed	30/52 (57.7%)	4/16 (25%)	0.0433
Shockable rhythm	32/102 (31.4%)	4/24 (16.7%)	0.2103
Process			
Response times EMS, min			
mean time to first vehicle on scene*	7.5 (5.6; 9.8)	9.1 (6.8; 12.1)	0.0347
mean time on scene*	34.2 (24.9; 41.9)	40.4 (20.4; 45.7)	0.8883
Arrest location outside city limits*	18/69 (26.1%)	9/24 (37.5%)	0.3063
Outcome			
Any ROSC	50/102 (49.0%)	8/24 (33.3%)	0.1808
Primary outcome on scene			
Declared dead on scene	35/102 (34.3%)	15/24 (62.5%)	0.019
Hospital admission			0.71
under ongoing CPR	24/67 (35.8%)	2/9 (22.2%)	
with ROSC	43/67 (64.2%)	7/9 (77.8%)	
Survival to hospital discharge			
of all cases admitted to hospital	27/67 (40.3%)	3/9 (33.3%)	1
of all cases	27/102 (26.5%)	3/24 (12.5%)	0.1885

^*﻿^without 2016. *n.a* not applicable, *OHCA* out-of-hospital cardiac arrest, *CA* cardiac arrest, *PEC* pre-existing conditions, *CPR* cardiopulmonary resuscitation, *EMS* emergency medical services, *ROSC* return of spontaneous circulation

Patients did not differ significantly regarding distribution in age and sex (*p* = 0.716 and *p* = 0.639 respectively). A pre-existing condition (PEC) with relevant impact on independent daily activities was documented more often in the pandemic period compared to the non-pandemic periods 66.7% (16/24) vs 38.2% (39/102), *p* = 0.020, while no PEC or a PEC with little impact on independent daily activities was documented less often, i.e. 20.8% (5/24) vs 50.0% (51/102),(*p* = 0.0117).

**Fig. 2:**
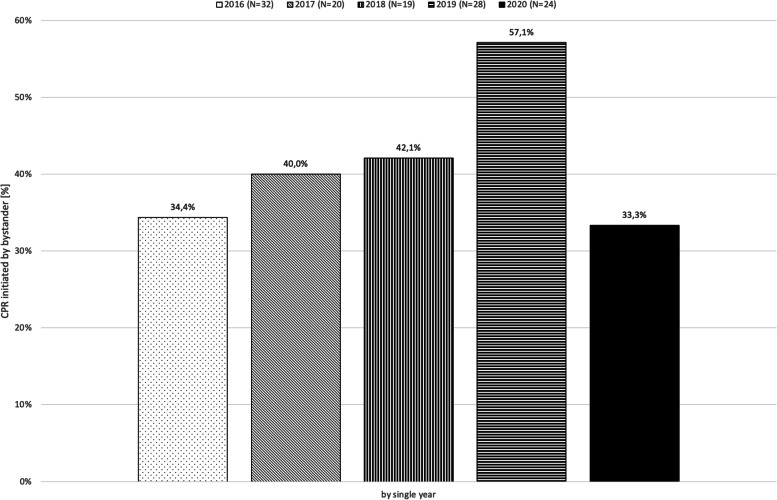
CPR initiated by bystander by year. The increase in 2019 compared to the other years was not statistically significant (*p* = 0.0799)

While in the non-pandemic period 52.5% (52/99, 3 unknown cases excluded) cases of OHCA were witnessed by bystanders, 66.7% (16/24) of cases were witnessed by bystanders during the pandemic period (*p* = 0.256). The rate of CPR provided by bystanders, which also included all non-medical emergency personnel as recommended by Utstein-style, was 43.4% (43/99) in the non-pandemic period versus 33.3% (8/24) in the pandemic period (*p* = 0.489, Fig. 2).

The by-year analysis for bystander CPR showed an increase in the rate of bystander CPR to 57.1% (16/28, 1 unknown case excluded) in 2019, followed by a drop to 33.3% (8/24) in 2020 (Fig. 2). Neither the increase in 2019 compared to the other years (*p* = 0.079), nor the drop in 2020 compared to 2019 proved to be statistically significant (*p* = 0.102).

In cases of witnessed CA, CPR was initiated by bystanders in 57.7% (30/52) of cases in the non-pandemic period and this rate decreased significantly to 25% (4/16) in the pandemic period (*p* = 0.043). Likewise but not significantly, the number of shockable rhythms decreased from 31.4% in the non-pandemic period (32/102) to 16.7% (4/24) in the pandemic period (*p* = 0.210).

EMS response times (i.e. time from call to first vehicle on scene) were significantly longer during the pandemic period compared to the non-pandemic period (*p* = 0.034), while time on scene did not differ significantly (*p* = 0.888, (Table 1). Return of spontaneous circulation (ROSC) at any time during CPR did not occur significantly less often in the pandemic period (non-pandemic: 49.0%, 50/102 vs 33.3%, 8/24; *p* = 0.180, Table 1). More resuscitation attempts were terminated on scene during the pandemic period (pandemic: 62.5% (15/24) vs non-pandemic: 34.3% (35/102); (Table 1 and Fig. 4), resulting in a smaller number of patients being transported to hospital with ongoing resuscitation attempts (*p* = 0.019). The state of those patients (ongoing CPR vs ROSC) at hospital admission did not differ significantly (*p* = 0.710).

SARS-CoV-2-infection status was not available for patients who were declared dead on scene during the pandemic period. Of the *N* = 9 patients admitted to a hospital during the pandemic period, 3/) were tested for SARS-CoV-2 with 1/3 resulting positive.

Survival to hospital discharge of all patients admitted to hospital did not significantly differ in between the analyzed periods (non-pandemic 40.3%, 27/67 vs. pandemic: 33.3%, 3/9; *p* = 1, (Table 1), just like survival to hospital discharge of all patients (non-pandemic: (26.5%, 27/102 vs pandemic: 12.5%, 3/24; *p* = 0.188).

Regarding further qualitative, infrastructural challenges to the rescue system, Supplementary Table 1 lists COVID-19 related changes. Supplementary Table 2 summarizes guidelines for an adjusted approach by EMS personnel, as issued by the Medical Head of the local EMS system.

A smartphone-based alerting system for trained rescuers (SAS) started in July 2018, had been implemented in July 2018 and was temporarily stopped from March 16th, 2020 until May 26th, 2020. It was reactivated following the provision of protective gear for all registered rescuers.

The out-of-hospital eCPR program was initiated in September 2019 and paused on March 18th, 2020 until July 1st, 2020. Apart from staff health protection, sparing of critical resources, i.e. extracorporeal circulation equipment which was reserved for possible in-hospital respiratory support, was another important reason.

According to the *Malteser Hilfsdienst* e.V., one of the providing organizations of basic life-support (BLS)-courses, training for laypeople was paused with the start of the lock-down. It was resumed June 1st, with a markedly changed curriculum, lower-yield educational methods and reduced course capacity (Supplementary Table 1). In the Federal State of Baden-Württemberg, 900 planned courses had to be cancelled due to the pandemic. In the same period in 2019, almost 12.000 laypeople were trained in First Aid and BLS [15]. Similar changes were seen by other course providers such as the *German Red Cross*.

Kids Save Lives activities were not specifically addressed in official regulations, but due to general distancing measures according to the *Ordinance of the State Government on Infection Protection Measures against the Spread of the SARS-CoV-2 Virus*, they are currently not applicable. (Supplementary Table 1).

Current interventions focus on protective gear and trainings for healthcare workers and volunteers to account for safety of all CPR providers.

## Discussion

This study provides a comprehensive quantitative and qualitative overview of changed proceedings in CPR across the whole chain of survival due to the COVID-19 pandemic in the Freiburg region. Quantitative data according to Utstein style from the prehospital and hospital admission phase shows only a moderate impact on bystander CPR rates, EMS response times and transportation rate after OHCA has been documented during the COVID-19 pandemic. Qualitative evaluation further reveals profound changes in the whole rescue system.

Major findings in this current investigation are longer mean EMS response times during the pandemic period. While our data do not provide a full explanation, longer turnover times per vehicle and case due to increased hygiene measures as well as staffing issues might have contributed to this observation, leading to less availability of ambulances and thus longer distances and travel times per unit. However, this remains speculative and warrants further investigation. Baldi et al. described a prolongation of 3 min in EMS arrival time, which they, at least in part, attributed to a higher case-load [8]. In two systematic reviews, prolonged EMS response times were consistent findings across several studies [16, 17].

Furthermore, there is a tendency towards lower rate of shockable rhythms and lower rates of ROSC during the pandemic period. This is also in line with the increased severity of “pre-existing conditions” (PEC) documented in the pandemic period, comparable with the recently published experience from Northern Italy and Paris [8, 9], as well as an increase in suspected non-cardiac causes of arrest.

The EMS documentation of „pre-existing conditions “(PEC) originally refers to comorbid and functional status with impact on independent living as the discriminator. However, it leaves room for a subjective evaluation by the emergency physician, based on the available information on scene. Hence, it does not sharply discriminate between chronic diagnoses and immediate peri-arrest illness and acute deterioration. More critical conditions at the time of cardiac arrest might have accounted for the shift to document worse PEC values characterizing the status preceding the event. Currently, this cannot be strengthened by additional data, and has to be speculated on.

In the literature, progressive deterioration of chronic conditions as well as delays in seeking medical attention and visits to the emergency department for acute, new-onset conditions such as acute coronary syndromes or severe infections have been described [18–23].

Beyond that, the data suggests a change in EMS decision making on scene during the pandemic period with less patients affected by OHCA were transported to hospital and more CPR attempts were terminated on scene (Fig. 4). Worse pre-existing conditions may have played a role in supporting these terminations of resuscitation efforts.

Also, the fear of EMS personnel of self-infection due to CPR during transportation might have contributed to this finding, but this was not examined. The odds of a favorable outcome may have further been decreased by the general changes in the rescue system described herein.

A key factor of favorable neurologic survival following OHCA is bystander CPR [24]. Before the pandemic, intensive efforts have been undertaken to improve the corresponding rates in Germany. Public campaigns and events, as well as the start of an app-based alarm system resulting in increased interest of the local and regional media in cardiac arrest, first aid, and related themes, supported this need in the Freiburg region in July 2018 leading to an increase in bystander CPR from 42.1 to 57% within 1 year. Although statistically not significant, the dynamic of the bystander CPR rate is remarkable regarding its drop back to 33% in 2020 after withholding the smartphone-based activation of first responders from March 18th due to public health and safety concerns. The bystander CPR rate of 2020 resembles the rates of 2016 through 2018 before the start of the app-based alarm system (Fig. 2). Another important driver of improvement in bystander-CPR rates, i.e. *Kids Save Lives* programs, has been paused together with the lock-down of schools. As these are struggling to return to normal routine, resuming CPR teaching activities poses further challenges on the system. A group of authors has therefore suggested a “Renewed Kids Save Lives campaign” to further increase awareness and fight sudden cardiac death in the era of COVID-19″ [25].

**Fig. 3:**
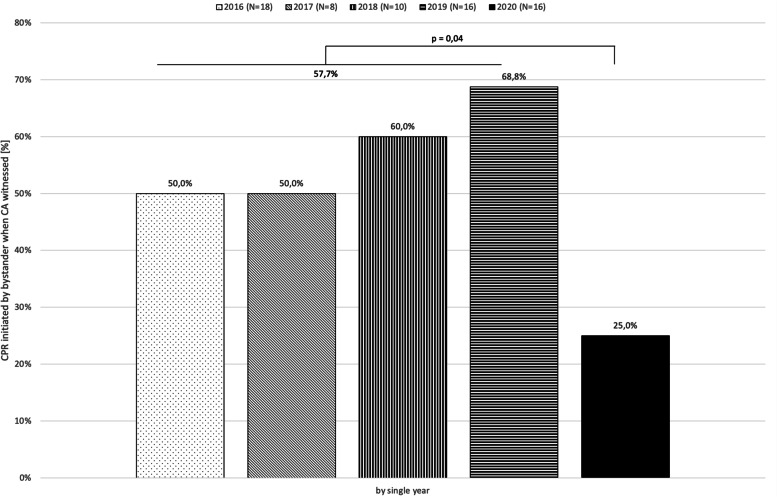
CPR initiated when CA witnessed by year

Furthermore, the possible impact of the pandemic on the behavior in OHCA of the public, i.e. patients, relatives, bystanders with their willingness to help and to call for help, has also to be taken into account. We observed a significant drop in bystander CPR rates during the pandemic, when the OHCA was directly witnessed (Fig. 3). This is again in line with findings in several studies, as summarized in the reviews by Lim [16] and Scquizzato [17]. A multinational, social media-based public survey by Grunau and coworkers confirmed fear of infection as a major obstacle to commence bystander CPR, among others [26]. It adds to the known barriers to perform CPR, such as fear of causing harm, or low confidence in own skills. Perceptions and possible fears regarding bystander CPR should be analyzed further in order to define suitable programs to inform the public during pandemic waves, and to undertake measures to preserve or improve these rates. Grunau et al’s results suggest that provision of protective gear has the potential to increase willingness to help. Further emphasizing compression-only CPR, or modifing the Basic Life Support algorithm by omitting the listen and feel-component of checks for breathing are further examples. Some national councils have included covering mouth and nose with tissues or face covers while performing CPR in their COVID-19 recommendations [11, 27]. Notably, our findings are in contrast to what we expected: In case of observed OHCA, the observers are often relatives or friends. We would expect these persons (probably knowing whether the relative is healthy or might suffer from COVID-19) are ready to help even under pandemic conditions. As very few people used to be around public places, we expected that the bystander CPR rate in all OHCA cases (including the non-observed OHCA cases) would have dropped, which it did not.

**Fig. 4:**
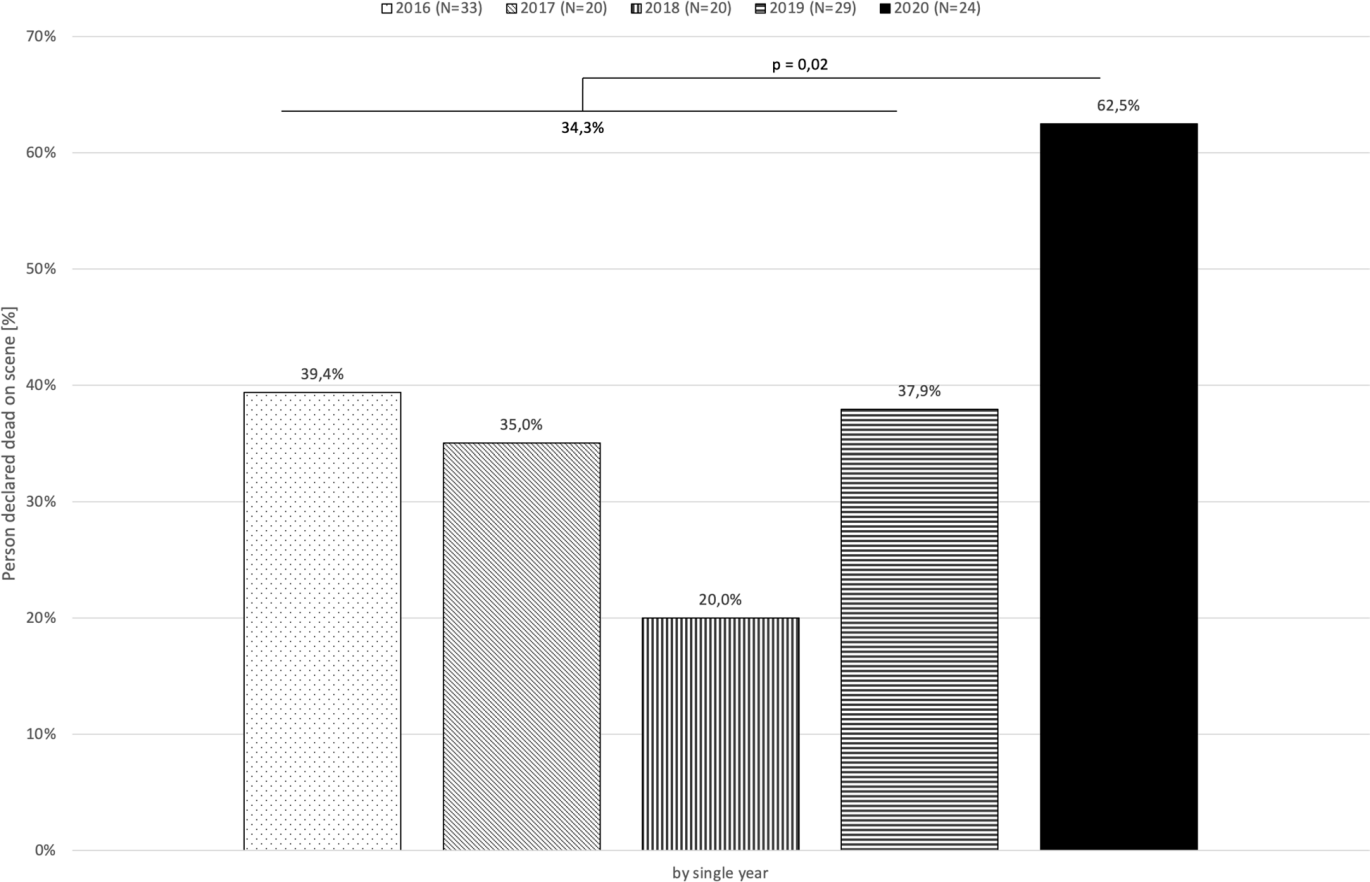
Patients declared dead on scene by year

The app-based alarm system was reactivated on May 26th, 2020 following the provision of protective gear for all registered rescuers. With the reduction of infection numbers following the general lock-down, it is planned to stepwise resume all additional resuscitation services. However, processes will still need to be adjusted to evolving evidence around infection risk through resuscitation measures. Hereby precautions for staff safety are a prerequisite. As all voluntary actions in the provision of CPR depend on the willingness and competence to help, perceptions and fears in the public need to be addressed with impactful educational measures. Although our data does not show a significant impact on survival, the overall survival after OHCA in 2020 halved compared to the same time periods in 2016 through 2019.

Public training in basic life support is another important pillar to enhance rates and quality of bystander CPR. The classic training formats had to be discontinued during the pandemic. Alternative digital formats with online parts and blended learning concepts should be further developed, adjusted to local needs and made easily accessible to the public, as a complement to reduced BLS training capacity. In their COVID-19 guidelines, the ERC therefore underlines the growing importance of “distance learning, self-directed learning, augmented and virtual learning” [10]. Birkun demonstrated a motivating effect of distance learning via a massive open online course regarding willingness to provide CPR, and a considerable increase in course registrations for this online course format [28]. Ali and colleagues systematically reviewed different CPR training strategies, including online-only-delivery, a feasible fallback-option when face-to-face training is not possible [29]. Virtual reality CPR training applications are increasingly being developed and made publicly available, facilitating low-budget-high-fidelity virtual training at home. Some of them have been designed in cooperation with national resuscitation councils, such as “Lifesaver VR” (Resuscitation Council UK, in English) [30], “TK-RescueMeVR” (German Resuscitation Council, in German) [31] or VR CPR (Italian Resuscitation Council) [32, 33]. While public institutions and healthcare authorities are struggling with more immediate effects of the pandemic and the disease itself, stakeholders in resuscitation care should be aware of all details and collateral effects on any part of the rescue system. Lay persons should be informed about infection risk when performing BLS and about measures to reduce the risk. Furthermore, it is very important to inform people about current recommendations (BLS under COVID conditions) to avoid that patients suffering from OHCA do not receive adequate measures. In line with ILCOR Consensus on Science and Treatment Recommendations (CoSTR) on improvement of system performance, we therefore took into account clinical outcome data, as well as system level variables [34] and qualitative information to inform dealing with the current pandemic wave, and foster preparedness for future pandemics with regard to cardiac arrest care.

### Limitations of our study

Cases of OHCA where no CPR was attempted were not included in this study. It is therefore possible that the incidence of OHCA was higher and survival lower. EMS dispatchers were instructed to investigate regarding symptoms of COVID-19. The impact of a warning category being assigned to a call on the process times was not analyzed. The COVID infection status of OHCA patients, if not reported by proxies or confirmed by laboratory testing in hospital was not automatically available at the time of arrest, nor retrospectively in all cases due to legal data protection limiting exchange between public health service and EMS. Ambulance process times of 2016 were not available and therefore not included in this study. Neurologic recovery is an important outcome in resuscitation studies. However our study was mainly focused on the prehospital period including hospital admission. Further data sources and outcome information are being prepared for future analyses.

### Generalizability

The generalizability of our study is limited due to the two-tiered, physician based nature of our rescue system. Regarding transport times, results from our urban area might be different from more rural areas. The additional services such as smartphone activated rescuers and eCPR are not available in other systems, so bystander CPR rates as well as transport with ongoing CPR might differ. Although relevantly affected, our area did not suffer from most severe strain as oher regions did, where caseload might be more pronounced. Thus, with higher or lower COVID-19 incidence or SARS-CoV-2 infection rates in other pandemic waves, or differently affected regions, outcomes and quantitative system level characteristics may vary. Therefore, muti-regional, national and international publications and review thereof are needed to achieve a more granular picture of pandemic related changes to resusciation systems worldwide, understand systematic phenomena and identify potential intervention targets.

## Conclusion

In the region of Freiburg, the COVID-19 pandemic has had a profound impact on the resuscitation landscape, along the whole chain of survival. Bystander CPR rates dropped, and innovative programs had to be discontinued, ultimately resulting in fewer patients being transported to a hospital after OHCA.

Although there fortunately has not been an effect of the pandemic on overall survival after OHCA yet, the authors believe that effective countermeasures have to be taken early to prevent these effects of becoming statistically significant. Current countermeasures in the city of Freiburg focus on protective gear and trainings for healthcare workers and volunteers to account for safety of all CPR providers. Furthermore, public educational campaigns and appeals should be employed to raise awareness for the importance of bystander CPR and hopefully ultimately improve bystander CPR-rates again.

## Supplementary Information

**Additional file 1.**

**Additional file 2.**

## Data Availability

The datasets used and/or analyzed during the current study are available from the corresponding author on reasonable request.
