# Supplementary material for: Observational study on implications of the COVID-19-pandemic for cardiopulmonary resuscitation in out-of-hospital cardiac arrest: qualitative and quantitative insights from a model region in Germany
Source: BMC Emerg Med. 2022 May 18;22:85. doi: 10.1186/s12873-022-00628-2 (PMC9116069; doi:10.1186/s12873-022-00628-2)
Supplement: Supplementary file 1 — Additional file 1. [file 12873_2022_628_MOESM1_ESM.docx]

Observational study on implications of the COVID-19-Pandemic for Cardiopulmonary Resuscitation in Out-of-Hospital Cardiac Arrest:

Qualitative and quantitative nsights from a Model Region in Germany

Domagoj Damjanovic^1§^, Jan-Steffen Pooth^1§^, Rebecca Steger^2^, Martin Boeker^3^, Michael Steger^2^, Julian Ganter^1^, Tobias Hack^1^, Klemens Baldas^4^, Paul Marc Biever^5^, Daniel Schmitz^4^, Hans-Jörg Busch^2^, Michael Patrick Müller^4^, Georg Trummer^1^, Bonaventura Schmid^2^

^§^These authors contributed equally to this work

**Supplementary Table 1: OHCA in COVID – Qualitative changes in resuscitation processes**

| **Feature of Rescue System** | **Covid-related changes** | **Reference*** | **Additional information/ Data source** |
| --- | --- | --- | --- |
| Recognition of cardiac arrests | Lock-down; more arrests occurring at home. | L | (1) |
| Call for help | Reluctance to seek medical attention even with critical or worsening conditions | L | (1) |
| Dispatch | Check and categorize possibility of infection (positively tested, infection possible or infection unlikely) prolongs time from call to dispatch  Inform on-scene teams regarding suspected infection status in real time | M | Local guidelines; see also supplementary table 2 |
| EMS response time | Prolonged | M, L | (1) |
| Changes in Procedures of EMS, use of PPE, staffing | Be alert  Check infection status  Part of team stays outside  Wear PPE (peer-monitored donning and doffing)  Radio ahead to receiving hospital | M | Local guidelines; see also supplementary table 2 |
| App-based trained rescuer activation | Stopped March 16^th^  Restarted May 26^th^ | M | Suffiicient PPE for all volunteers provided |
| Bystander-willingness | Reduced due to fear of infection | L | (2)  To be evaluated with additional survey |
| BLS trainig for laypeople | Stopped March 18^th^  Restarted June 1^st^  Less training capacity, reduced number of participants  Lower-yield educational methods (explanation) instead of demonstration | M  L | Malteser starten wieder mit Erste-Hilfe-Ausbildung: Kurse an Vorgaben des Infektionsschutzes angepasst [Internet]. Malteser in Deutschland. [cited 2021 May 29]. Available from: https://www.malteser-bw.de/wir-malteser/presse-und-medien/news-detailansicht/news/malteser-starten-wieder-mit-erste-hilfe-ausbildung-kurse-an-vorgaben-des-infektionsschutzes-angepass.html  According to internal guidance for BLS instructors; available upon reasonable request |
| Prehospital eCPR | Stopped March 18^th^  Restarted July 1^st^ | M | PPE added  Modified approach according to guidelines issued for EMS, see also supplementary table 2 |
| Resuscitation Research | Several projects on hold  Before-after-before constellation in some projects (which resembles a *natural experiment*)  Reporting of COVID-19 infection status of cardiac arrest patients |  | Adding COVID19 information  German Resuscitation Registry has added guidelines how to insert information on infection status in existing data entry templates  New information source (public health departments) needs to be integrated, potentially requires adjustments of data protection aspects |
| Federal State-wide Kids Save Lives program *„Löwen Retten Leben“* | Program not officially stopped but *de facto* currently  not applicable in its original form due to general distancing measures according to the *Ordinance of the State Government on Infection Protection Measures against the Spread of the SARS-CoV-2 Virus* | M |  |

EMS: Emergency Medical Services; PPE: Personal protective equipment; eCPR: extracorporeal CPR; *Reference: Description of the supporting information: M supported by qualitative or quantitative results, as shown in the mansucript; L reference from the literature; A reasonable assumption

References

1. Baldi E, Sechi GM, Mare C, Canevari F, Brancaglione A, Primi R, et al. COVID-19 kills at home: the close relationship between the epidemic and the increase of out-of-hospital cardiac arrests. Eur Heart J [Internet]. 2020 Jun 20 [cited 2020 Jan 7];(ehaa508). Available from: https://doi.org/10.1093/eurheartj/ehaa508

2. Grunau B, Bal J, Scheuermeyer F, Guh D, Dainty KN, Helmer J, et al. Bystanders are less willing to resuscitate out-of-hospital cardiac arrest victims during the COVID-19 pandemic. Resusc Plus. 2020 Dec 1;4:100034.
