# Supplementary material for: Observational study on implications of the COVID-19-pandemic for cardiopulmonary resuscitation in out-of-hospital cardiac arrest: qualitative and quantitative insights from a model region in Germany
Source: BMC Emerg Med. 2022 May 18;22:85. doi: 10.1186/s12873-022-00628-2 (PMC9116069; doi:10.1186/s12873-022-00628-2)
Supplement: Supplementary file 2 — Additional file 2. [file 12873_2022_628_MOESM2_ESM.docx]

Implications of the COVID-19-Pandemic for Cardiopulmonary Resuscitation in Out-of-Hospital Cardiac Arrest:

Insights from a Model Region in Germany

Domagoj Damjanovic^1§^, Jan-Steffen Pooth^1§^, Rebecca Steger^2^, Martin Boeker^3^, Michael Steger^2^, Julian Ganter^1^, Tobias Hack^1^, Klemens Baldas^4^, Paul Marc Biever^5^, Daniel Schmitz^4^, Hans-Jörg Busch^2^, Michael Patrick Müller^4^, Georg Trummer^1^, Bonaventura Schmid^2^

**Supplementary Table 2: Guidelines for EMS personnel, Covid19-related modifications of approach to emergencies**

| **Task** | **Covid-related changes** | **Additional information** |
| --- | --- | --- |
| Approach to scene, infection control precautions | Level 1, Basic   - Surgical mask for staff in all patient encounters - Physical distancing as far as possible - Not touching own face - Peer monitoring   Level 2, SARS-CoV-2-infection *possible*   - Level 1 *plus* - Reduce number of team members with immediate patient contact - Surgical mask for patient as early as possible - Peer monitored doffing - In close patient contact: precautions as in Level 3 - Peer monitored donning   Level 3, SARS-CoV-2-infection *confirmed*   - Level 2 *plus* - FFP2-mask, safety goggles - 2 pairs of gloves - Long Sleeve gown | - Initial situational check and triage by experienced team member, stratification regarding infection status, definition of Level of precautions - Radio to team members - Reduce all measures to necessary minimum - Donned team member stays with patient, additional team members only if necessary - Encourage patients‘ self extrication and mobility as reasonable - Consider stepping down vehicle class for transport after on-scene intervention and stabilization |
| Respiratory failure | - Avoid aerosol therapy in confined spaces/ ambulance vehicle - Avoid non-invasive ventilation via face-mask - Avoid airway suctioning; if necessary use goggles |  |
| Endotracheal Intubation | - Don PPE: Gown, FFP2 mask, safety goggles - Reduce manpower in immediate area - Avoid intermediate bag-mask-ventilations if possible - Use video-laryngoscope - Use viral filter in breathing circuit close to patient - Do not allow for accidental disconnections in breathing circuit - In CPR: aim for early and rapid endotracheal intubation |  |
| CPR | - Don PPE: Gown, FFP2 mask, safety goggles - Reduce manpower in immediate area - On recognition of peri-arrest situation, don PPE as early as possible - Time delays in start of CPR due to hygiene precautions must be accepted - Keep distance to patient, especially facial region, as much as possible (e.g. in check for breathing) - Apply NRB oxygen mask as aerosol- and droplet protection plus compression-only CPR until BMV available - Hierarchy of airway managemenr in protection level: ETI>SGA>BMV>unprotected airway - Use viral filters in breathing circuit - Consider ETI early - No chest comressions during laryngoscopy - Whenever possible, use videolaryngoscopy - Block ETT cuff sufficiently - Connect breathing circuit securely - Consider mechanical CPR device early- this supports distancing |  |
| Patient transport | - Except in children, no relatives can be accepted inside the ambulance during transport |  |
